# Diagnostic dilemma’s in the new world of ICD-11 personality disorders

**DOI:** 10.1192/j.eurpsy.2021.175

**Published:** 2021-08-13

**Authors:** M. Wise

**Affiliations:** Psychiatry, Brent CMHT, London, United Kingdom

**Keywords:** ICD-11, Personality Disorder, personality disorder

## Abstract

Personality disorders have ever been a troublesome group. From the early 90’s ICD 10 tidied up the group. DSM-IV, IV-TR, aand then DSM 5, changed the style but not substance, leaving clinicians to grapple with thorny questions of multiple diagnoses, treatment and prognosis. International views on the utility of the diagnosis often depended upon the institution or the funding mechanism. Were fears of exclusion and stigma dominated or where there was no treatment, there was under-diagnosis, such as in the United Kingdom and the Republic of Ireland. Where a label was a ticket of entry to treatment and funding, diagnostic generosity prevailed, such as in Australia, New Zealand and the United States. Gender discrepancies disappeared with structured interviews, and interest grew in the category which seem to only include the most severe forms. For many years the DSM taskforce tried to shift the construct but shied away from the cliff edge; a bold new initiative did not materialise. It was left to the ICD-11 to generate a much more adventurous and positive view of how characterological traits shift under pressure, moving from something that may at first have helped patients to ‘survive’ to something that became maladaptive and harmful. With a court tested case Dr Wise will demonstrate the differences between ICD-10 and ICD-11 highlighting the more important differences: onset, course and severity descriptors. PD’s are no longer lifelong impairments. Prepare for ‘The shock of the new’!

**Disclosure:**

No significant relationships.

